# Breastfeeding and childhood cancer

**DOI:** 10.1054/bjoc.2001.2110

**Published:** 2001-11

**Authors:** U.K. Childhood Cancer Study Investigators

**Affiliations:** V Beral, NT Fear, F Alexander and P Appleby. A complete list of investigators is given in: The United Kingdom Childhood Cancer Study: objectives material and methods. Br J Cancer 2000; 82(5): 1073–1102

**Keywords:** breastfeeding, childhood cancer

## Abstract

The relationship between childhood cancer and having been breastfed in infancy was investigated in the UK Childhood Cancer Study (UKCCS), a national, population-based case-control study. Analyses included 3500 children with cancer (cases) of whom 1637 were diagnosed with leukaemia, 114 with Hodgkin's disease, 228 with non-Hodgkin's lymphoma and 1521 with other cancer and 6964 controls. 62% cases and 64% controls were reported to have ever been breastfed. There was weak evidence, of borderline statistical significance, that having been breastfed was associated with a small reduction in the odds ratios for leukaemia (odds ratio = 0.89, 95% Cl 0.80–1.00, *P* = 0.06), and for all cancers combined (odds ratio = 0.92, 95% Cl 0.84–1.00, *P* = 0.05). Combining data from the UKCCS with results from other published studies showed a small reduction in the odds ratios for leukaemia, Hodgkin's disease, non-haematological cancers, and all childhood cancers combined, associated with ever having been breastfed. It is unclear whether the apparent small reduction in the odds ratio for these various types of childhood cancer is a generalized effect of breastfeeding or whether it reflects some systematic bias in the majority of studies that have investigated the question. © 2001 Cancer Research Campaign http://www.bjcancer.com


*British Journal of Cancer* (2001) **85**(11), 1685–1694
© 2001 Cancer Research Campaign
doi: 10.1054/ bjoc.2001.2110, available online at http://www.idealibrary.com on IDEAL

### Breastfeeding and childhood cancer

**UK Childhood Cancer Study Investigators***

*V Beral, NT Fear, F Alexander and P Appleby. A complete list of investigators is given in: The United Kingdom Childhood Cancer Study: objectives, material, and methods. Br J Cancer 2000; 82(5): 1073–1102

**Summary** The relationship between childhood cancer and having been breastfed in infancy was investigated in the UK Childhood Cancer Study (UKCCS), a national, population-based case-control study. Analyses included 3500 children with cancer (cases) of whom 1637 were diagnosed with leukaemia, 114 with Hodgkin's disease, 228 with non-Hodgkin's lymphoma and 1521 with other cancer and 6964 controls. 62% cases and 64% controls were reported to have ever been breastfed. There was weak evidence, of borderline statistical significance, that having been breastfed was associated with a small reduction in the odds ratios for leukaemia (odds ratio = 0.89, 95% CI 0.80–1.00, $P = 0.06$), and for all cancers combined (odds ratio = 0.92, 95% CI 0.84–1.00, $P = 0.05$). Combining data from the UKCCS with results from other published studies showed a small reduction in the odds ratios for leukaemia, Hodgkin's disease, non-haematological cancers, and all childhood cancers combined, associated with ever having been breastfed. It is unclear whether the apparent small reduction in the odds ratio for these various types of childhood cancer is a generalized effect of breastfeeding or whether it reflects some systematic bias in the majority of studies that have investigated the question. © 2001 Cancer Research Campaign http://www.bjcancer.com

**Keywords**: breastfeeding; childhood cancer

The identification of leukaemia and lymphoma viruses in various animal species (Gross, 1951; Vogt and Ishizaki, 1965) prompted the search for viral causes of human leukaemia and lymphoma. The Epstein Barr virus has been associated with Burkitt's lymphoma in children (Epstein et al, 1964) and the human T-cell leukaemia virus with a rare form of adult leukaemia/lymphoma (Poiesz et al, 1980) but, as yet, no specific infectious agent has been associated with the majority of human leukaemias or lymphomas. Recently, attention has focused on the association between surrogate markers of exposure to infectious agents and the risk of childhood leukaemia (Greaves and Alexander, 1993; Kinlen, 1995).

Breastfed infants are at a reduced risk of gastrointestinal and respiratory infections (WHO Collaborative Study Team on the Role of Breastfeeding on the Prevention of Infant Mortality, 2000), but are also at an increased risk of mother-to-child transmission of certain infectious agents (Ruff, 1994; Nduati et al, 2000). Several case-control studies of children with leukaemia have reported a significantly reduced risk associated with having been breastfed as infants (Shu et al, 1999; Smulevich et al, 1999; Infante-Rivard et al, 2000; Bener et al, 2001), although not all studies have noted such a relationship (van Steensel-Moll et al, 1986; McKinney et al, 1987; Davis et al, 1988; Magnani et al, 1988; Shu et al, 1995; Petridou et al, 1997; Dockerty et al, 1999; Schüz et al, 1999; Rosenbaum et al, 2000; Hardell and Dreifaldt, 2001). Others have reported a significantly reduced risk of Hodgkin's disease (Grufferman et al, 1998) and non-Hodgkin's lymphoma (Bener et al, 2001) in breastfed children.

We report here the results of an analysis of breastfeeding patterns in infancy among children with leukaemia and other cancers and among control children in the UK Childhood Cancer Study (UK Childhood Cancer Study Investigators, 2000). An a priori hypothesis was that any association with breastfeeding should be seen for childhood leukaemia, and possibly for childhood lymphoma, but not for other childhood cancers.

### MATERIALS AND METHODS

### Subjects

The UKCCS is a national population-based case-control study. Details of the study design are given elsewhere (UK Childhood Cancer Study Investigators, 2000). Briefly, children aged 0–14 with cancer diagnosed in England and Wales (1992–96) and Scotland (1991–94) were eligible for inclusion in the study. Detailed diagnostic review information was obtained from multiple sources, including histopathology reviews and clinical trial data. Subtyping of leukaemia was made possible by collecting together and reviewing cytogenetic and molecular details (UK Childhood Cancer Study Investigators, 2000). For each case, two controls were randomly selected from Family Health Services Authorities (in England and Wales) and Health Boards (in Scotland) matched to the case by month and year of birth, sex and region of residence at diagnosis. Eligible controls who did not participate in the study were replaced by another matched control until, where possible, two controls had been enrolled for each participating case. Participation rates were 87% for cases and 72% for first-choice controls.

The mothers of 3838 cases and 7629 controls were interviewed, using a standard questionnaire, that included questions about social, medical and behavioural factors. Included among the questions to the mothers were 'Did you ever breastfeed (the child)? How old was (the child) when you gave your last breast feed? Did you ever use formula milk? If so, how old was (the child) when he/she had his/her first

*Received 18 June 2001*
*Revised 8 August 2001*
*Accepted 9 August 2001*

*Correspondence to:* V Beral,Imperial Cancer Research Fund Cancer Epidemiology Unit, Gibson Building, Radcliffe Infirmary, Woodstock Road, Oxford OX2 6HE, UK

*See Appendix for Management Committee, Regional Investigators and Data Management Processing Group formula feed? Was (the child) ever given expressed milk from a milk bank?' Responses to these questions are examined in this report.

### Analysis

A total of 943 children aged under 12 months at diagnosis/pseudodiagnosis were excluded from the analyses to prevent the introduction of possible biases if breastfeeding were to cease prematurely in children aged under 12 months with cancer. A further 60 children were excluded because information about breastfeeding had not been provided by their biological mother. Overall 338 cases and 665 controls were excluded leaving 3500 cases and 6964 controls – 91% of all interviewed and potentially eligible subjects.

Analyses were carried out for all cancers combined (3500 cases), and separately for leukaemia (1637 cases), Hodgkin's disease (114 cases), non-Hodgkin's lymphoma (228 cases) and all other cancers (1521 cases). For certain analyses children with leukaemia were subdivided into those with acute lymphoblastic leukaemia (ALL, 1401 cases) and acute myeloid leukaemia (AML, 214 cases). Those with ALL were subclassified further, as having common ALL (cALL, 879 cases), T cell ALL (134 cases), and those with B-lineage ALL were further subclassified as TEL-AML 1 (95 cases) or hyperdiploidy (358 cases).

Since the hypotheses concerning the role of infectious agents in childhood cancer are chiefly concerned with leukaemia and lymphoma, and because of the possible differential participation of the parents of children with and without cancer and differential recall of events in childhood, some analyses were restricted to children with cancer. In these restricted analyses, the breastfeeding histories of children with leukaemia or lymphoma were compared with those of children with other cancers, i.e. taking the children with other cancers as 'controls'.

Odds ratios (ORs) and their 95% confidence intervals (CIs) were estimated using unconditional logistic regression (StataCorp, 1999) and adjusted for region (10 in total), sex, age at diagnosis (in single years), birth order (1, 2, 3, 4+) and deprivation index (7 levels). For certain analyses, additional adjustments were performed for the mother's age at leaving school and at the time of the child's birth, child's birthweight, whether or not the child had been admitted to a special care baby unit, and had attended day care in the first year of life, and maternal smoking when the child was 1 year old. Trends were assessed using likelihood ratio tests.

Results showing data from more than one study are presented in the form of plots of odds ratios. Each odds ratio is plotted as a black square, the area of which is proportional to the amount of statistical information for that particular estimate, i.e. the reciprocal of the variance of the log odds ratio, which reflects the reliability with which the odds ratio is estimated. The corresponding 95% CIs are shown as horizontal black lines. Where CIs are so narrow as to be contained entirely in the black box, they are plotted as white lines. Summary odds ratios are plotted in the form of a diamond, the width of which indicates the corresponding 95% CIs, and the dotted vertical line indicates the value of the summary odds ratio.

### RESULTS

Table 1 shows the distribution of children with leukaemia, Hodgkin's disease, non-Hodgkin's lymphoma, other cancers, all cancers and controls, according to whether or not they were reported to have ever been breastfed and the reported duration that they had been breastfed; it also shows the corresponding adjusted odds ratios, with 95% confidence intervals, and the results of tests for trend by duration of breastfeeding. About two thirds of the children were reported to have been breastfed, with almost one fifth for longer than 6 months. The adjusted odds ratios associated with ever having been breastfed were below 1.0 and of borderline statistical significance for leukaemia (OR = 0.89, 95%CI 0.80–1.00, $P$ = 0.06) and for all cancers combined (OR = 0.92, 95% CI 0.84–1.00, $P$ = 0.05). For each cancer type shown in Table 1, none of the trends with increasing duration of breastfeeding was statistically significant. The odds ratios in Table 1 were not materially altered after additional adjustment for maternal education, maternal age, birthweight, admission to a special care baby unit, attendance at day care, or maternal smoking.

**Table 1** Distribution of children with cancer and controls, according to cancer type and breastfeeding history, and the associated odds ratios* (ORs) and 95% confidence intervals (CIs)

| | Leukaemia *n* = 1637 | Hodgkin's disease *n* = 114 | Non-Hodgkin's lymphoma *n* = 228 | Other cancers *n* = 1521 | All cancers *n* = 3500 | Controls *n* = 6964 |
|---|---|---|---|---|---|---|
| a) Number (%) | | | | | | |
| never breastfed | 629 (38.4) | 43 (37.7) | 79 (34.7) | 592 (38.9) | 1343 (38.4) | 2500 (35.9) |
| ever breastfed | 1008 (61.6) | 71 (62.3) | 149 (65.4) | 929 (61.1) | 2157 (61.6) | 4464 (64.1) |
| duration < 1 month | 251 (15.3) | 23 (20.2) | 33 (14.5) | 241 (15.8) | 548 (15.7) | 1015 (14.6) |
| duration 1–6 months | 454 (27.7) | 29 (25.4) | 75 (32.9) | 389 (25.6) | 947 (27.1) | 2034 (29.2) |
| duration ≥ 7 months | 302 (18.5) | 19 (16.7) | 41 (18.0) | 298 (19.6) | 660 (18.9) | 1410 (20.3) |
| unknown | 1 (0.1) | – | – | 1 (0.1) | 2 (0.1) | 5 (0.1) |
| b) ORs* (95% CIs) | | | | | | |
| never breastfed (reference) | 1.0 | 1.0 | 1.0 | 1.0 | 1.0 | |
| ever breastfed | 0.89 (0.80–1.00) | 1.01 (0.67–1.53) | 1.03 (0.77–1.38) | 0.90 (0.80–1.02) | 0.92 (0.84–1.00) | |
| duration < 1 month | 0.96 (0.81–1.14) | 1.50 (0.88–2.57) | 1.04 (0.68–1.59) | 1.01 (0.86–1.20) | 1.01 (0.89–1.14) | |
| duration 1–6 months | 0.88 (0.77–1.02) | 0.85 (0.51–1.40) | 1.12 (0.80–1.50) | 0.83 (0.72–0.96) | 0.88 (0.79–0.98) | |
| duration ≥ 7 months | 0.85 (0.73–1.00) | 0.90 (0.50–1.60) | 0.90 (0.60–1.34) | 0.93 (0.79–1.08) | 0.89 (0.79–1.01) | |
| c) Test for trend | | | | | | |
| with duration of breastfeeding+ | $\chi_1^2$ = 1.11 $P$ = 0.29 | $\chi_1^2$ = 1.22 $P$ = 0.27 | $\chi_1^2$ = 1.07 $P$ = 0.30 | $\chi_1^2$ = 0.10 $P$ = 0.75 | $\chi_1^2$ = 1.50 $P$ = 0.22 | |

*Adjusted by age at diagnosis, sex, region, birth order and deprivation index. +Excludes never breast fed and unknown duration categories, based on median duration within each duration category (9 days, 91 days and 304 days) for the control population.

 *© 2001 Cancer Research Campaign*

Table 2 shows results, for various subtypes of leukaemia, for children who had ever versus never been breastfed and according to the reported duration of breastfeeding. The numbers in each category are comparatively small and none of the odds ratios or tests for trend with duration of breastfeeding was statistically significant. The results for leukaemia were also examined separately for first born children and the odds ratio associated with ever having been breastfed in this subgroup was 0.98 (95% CI 0.82–1.18).

Table 3 shows age-specific results according to whether or not the children were breastfed, separately for children with

**Table 2** Distribution of children with leukaemia, according to the type of leukaemia and breastfeeding history, and the associated odds ratios* (ORs) and 95% confidence intervals (CIs)

| | **Acute myeloid leukaemia (AML) *n* = 214** | **Acute lymphoblastic leukaemia (ALL) *n* = 1401** | **Sub-categories of acute lymphoblastic leukaemia** | | | |
|---|---|---|---|---|---|---|
| | | | **T cell ALL *n* = 134** | **Common ALL *n* = 879** | **Sub-categories of B-lineage ALL** | |
| | | | | | **TEL-AML 1 *n* = 95** | **Hyperdiploidy *n* = 358** |
| a) Number (%) | | | | | | |
| never breastfed | 92 (43.0) | 529 (37.8) | 48 (35.8) | 320 (36.4) | 34 (35.8) | 131 (36.6) |
| ever breastfed | 122 (57.0) | 872 (62.2) | 86 (64.2) | 559 (63.6) | 61 (64.2) | 227 (63.4) |
| duration < 1 month | 29 (13.6) | 217 (15.5) | 15 (11.2) | 149 (17.0) | 13 (13.7) | 64 (17.9) |
| duration 1–6 months | 61 (28.5) | 388 (27.7) | 43 (32.1) | 243 (27.7) | 23 (24.2) | 100 (27.9) |
| duration ≥ 7 months | 32 (15.0) | 266 (19.0) | 28 (20.9) | 167 (18.9) | 25 (26.3) | 63 (17.6) |
| unknown | – | 1 (0.1) | – | – | – | – |
| b) ORs* (95% CIs) | | | | | | |
| never breastfed (reference) | 1.0 | 1.0 | 1.0 | 1.0 | 1.0 | 1.0 |
| ever breastfed | 0.78 (0.58–1.05) | 0.91 (0.81–1.04) | 1.14 (0.78–1.66) | 0.96 (0.82–1.11) | 0.97 (0.62–1.50) | 0.96 (0.76–1.22) |
| duration < 1 month | 0.82 (0.53–1.26) | 0.98 (0.82–1.17) | 0.86 (0.47–1.55) | 1.09 (0.88–1.34) | 0.87 (0.45–1.67) | 1.14 (0.83–1.57) |
| duration 1–6 months | 0.85 (0.60–1.20) | 0.90 (0.77–1.04) | 1.27 (0.82–1.95) | 0.91 (0.76–1.09) | 0.80 (0.46–1.38) | 0.93 (0.71–1.23) |
| duration ≥ 7 months | 0.65 (0.43–1.00) | 0.89 (0.75–1.05) | 1.20 (0.73–1.97) | 0.92 (0.75–1.13) | 1.31 (0.76–2.25) | 0.87 (0.63–1.19) |
| c) Test for trend | | | | | | |
| with duration | $\chi_1^2 = 1.46$ | $\chi_1^2 = 0.50$ | $\chi_1^2 = 0.29$ | $\chi_1^2 = 0.52$ | $\chi_1^2 = 2.72$ | $\chi_1^2 = 1.47$ |
| of breastfeeding[+] | $P = 0.23$ | $P = 0.48$ | $P = 0.59$ | $P = 0.47$ | $P = 0.10$ | $P = 0.23$ |

*Adjusted by age at diagnosis, sex, region, birth order and deprivation index. [+]Excludes never breast fed and unknown duration categories, based on median duration within each duration category (9 days, 91 days and 304 days) for the control population.

**Table 3** Age-specific distribution of children with cancer and controls, and associated odds ratios* (ORs) and 95% confidence intervals (CIs)

| | **Leukaemia** | **Hodgkin's disease** | **Non-Hodgkin's lymphoma** | **Other cancers** | **All cancers** | **Controls** |
|---|---|---|---|---|---|---|
| Age 1 year | | | | | | |
| a) Numbers (%) | | | | | | |
| never breastfed | 59 (44.0) | – | 2 (66.7) | 67 (32.5) | 128 (37.3) | 261 (37.9) |
| ever breastfed | 75 (56.0) | – | 1 (33.3) | 139 (67.5) | 215 (67.7) | 427 (62.1) |
| b) ORs* (CIs) for ever | | | | | | |
| vs never breastfed | 0.76 (0.51–1.12) | – | 1.76 (0.09–35.9) | 1.48 (1.04–2.09) | 1.12 (0.84–1.48) | |
| c) *P* value (ever vs never) | 0.17 | – | 0.71 | 0.03 | 0.44 | |
| Ages 2–5 years | | | | | | |
| a) Numbers (%) | | | | | | |
| never breastfed | 332 (37.3) | 8 (53.3) | 20 (30.8) | 236 (42.9) | 596 (39.2) | 1088 (36.1) |
| ever breastfed | 558 (62.7) | 7 (46.7) | 45 (69.2) | 314 (57.1) | 924 (60.8) | 1926 (63.9) |
| b) ORs* (CIs) for ever | | | | | | |
| vs never breastfed | 0.95 (0.81–1.11) | 0.57 (0.19–1.69) | 1.24 (0.71–2.15) | 0.75 (0.62–0.91) | 0.88 (0.79–1.00) | |
| c) *P* value (ever vs never) | 0.52 | 0.31 | 0.45 | 0.004 | 0.05 | |
| Age 6+ years | | | | | | |
| a) Numbers (%) | | | | | | |
| never breastfed | 238 (38.8) | 35 (35.4) | 57 (35.6) | 289 (37.8) | 619 (37.8) | 1151 (35.3) |
| ever breastfed | 375 (61.2) | 64 (64.7) | 103 (64.4) | 476 (62.2) | 1018 (62.2) | 2111 (64.7) |
| b) ORs* (CIs) for ever | | | | | | |
| vs never breastfed | 0.85 (0.70–1.02) | 1.12 (0.71–1.75) | 0.99 (0.70–1.41) | 0.92 (0.77–1.09) | 0.91 (0.80–1.04) | |
| c) *P* value (ever vs never) | 0.09 | 0.62 | 0.97 | 0.34 | 0.16 | |

*Adjusted by age of diagnosis, sex, region, birth order and deprivation index.

© 2001 Cancer Research Campaign 

leukaemia, Hodgkin's disease, non-Hodgkin's lymphoma, other cancers, and all cancers combined. The divisions by age were determined a priori, with the specific hypothesis that the effects of any infectious agent would be greatest for cALL at ages 2–5 (Greaves and Alexander, 1993). For leukaemia the odds ratios did not differ significantly from 1.0 at any age and there is no statistical evidence that the results varied according to age at diagnosis. Most children with leukaemia were diagnosed at ages 2–5 years and in this age group the odds ratios for the various leukaemia subtypes for ever having been breastfed versus never having been breastfed were: 0.95 (0.80–1.12) for ALL; 0.95 (0.55–1.64) for AML; 1.03 (0.83–1.25) for cALL; 1.18 (0.64–2.18) for T cell ALL; 0.84 (0.50–1.40) for TEL-AML 1; and 1.03 (0.77–1.37) for hyperdiploidy. Thus there was no strong evidence of heterogeneity between the subtypes at those ages. Nor was there evidence that the findings for the lymphomas varied by age, although there was some evidence of heterogeneity in all other cancer categories. Detailed examination for specific cancers within this category revealed no consistent pattern. Of these, the most common cancers before the age of 6 were tumours of the central nervous system (57 cases at age 1, OR = 1.79, 95% CI 0.94–3.41; and 210 cases at age 2–5, OR = 0.91, 95% CI 0.67–1.23) and renal tumours (39 cases at age 1, OR = 1.85, 95% CI 0.85–4.02 and 99 cases at age 2–5, OR = 0.68, 95% CI 0.44–1.04). Both these tumour types contributed to the comparatively high odds ratio for children aged 1 and the comparatively low odds ratio at ages 2–5, and these may well be chance findings.

Table 4 shows results according to whether or not the child was ever given formula feed and, if so, the age the child was when such feeds were introduced. No consistent pattern is evident, and no results are statistically significant. Nor did the risk of childhood cancer appear to be influenced by having been given expressed milk from a milk bank. The results are, however, based on small numbers (about 2% of children ever received milk from a milk bank), the respective odds ratios were: 0.89 (0.57–1.39) for leukaemia; 1.50 (0.46–4.95) for Hodgkin's disease; 1.17 (0.46–2.93) for non-

**Table 4** Distribution of children with cancer and controls, according to the type of cancer and age at introduction of first formula feed, and the associated odds ratios* (ORs) and 95% confidence intervals (CIs)

| | **Leukaemia** *n* **= 1637** | **Hodgkin's disease** *n* **= 114** | **Non-Hodgkin's lymphoma** *n* **= 228** | **Other cancers** *n* **= 1521** | **All cancers** *n* **= 3500** | **Controls** *n* **= 6964** |
|---|---|---|---|---|---|---|
| a) Number (%) | | | | | | |
| no formula feeds | 223 (13.6) | 16 (14.0) | 32 (14.0) | 216 (14.2) | 487 (13.9) | 1047 (15.0) |
| first use <1 month | 1024 (62.6) | 73 (64.0) | 135 (59.2) | 960 (63.1) | 2192 (62.6) | 4136 (59.4) |
| first use ≥ 1 month | 390 (23.8) | 25 (21.9) | 61 (26.8) | 345 (22.7) | 821 (23.5) | 1778 (25.5) |
| unknown | – | – | – | – | – | 3 (<0.1) |
| b) ORs* (95% CIs) | | | | | | |
| no formula feeds | 1.0 | 1.0 | 1.0 | 1.0 | 1.0 | |
| first use <1 month | 1.11 (0.94–1.31) | 1.28 (0.72–2.26) | 1.22 (0.82–1.84) | 1.09 (0.92–1.29) | 1.11 (0.98–1.26) | |
| first use ≥ 1 month | 0.98 (0.81–1.18) | 1.11 (0.58–2.11) | 1.30 (0.83–2.03) | 0.93 (0.77–1.12) | 0.98 (0.86–1.13) | |
| c) *P* value (ever vs never) | *P* = 0.44 | *P* = 0.48 | *P* = 0.27 | *P* = 0.65 | *P* = 0.28 | |

*Adjusted by age at diagnosis, sex, region, birth order and deprivation index.

**Table 5** Distribution of children with cancer, according to cancer type and breastfeeding history, and the associated odds ratios* (ORs) and 95% confidence intervals (CIs) for leukaemia and lymphoma compared to all other cancers

| | **Leukaemia** *n* **= 1637** | **Hodgkin's disease** *n* **= 114** | **Non-Hodgkin's lymphoma** *n* **= 228** | **Other cancers** *n* **= 1521** |
|---|---|---|---|---|
| a) Number (%) | | | | |
| never breastfed | 629 (38.4) | 43 (37.7) | 79 (34.7) | 592 (38.9) |
| ever breastfed | 1008 (61.6) | 71 (62.3) | 149 (65.4) | 929 (61.1) |
| duration < 1 month | 251 (15.3) | 23 (20.2) | 33 (14.5) | 241 (15.8) |
| duration 1–6 month | 454 (27.7) | 29 (25.4) | 75 (32.9) | 389 (25.6) |
| duration ≥ 7 months | 302 (18.5) | 19 (16.7) | 41 (18.0) | 298 (19.6) |
| unknown | 1 (0.1) | – | – | 1 (0.1) |
| b) ORs* (95% CIs) | | | | |
| never breastfed (reference) | 1.0 | 1.0 | 1.0 | |
| ever breastfed | 1.00 (0.85–1.17) | 1.24 (0.79–1.95) | 1.26 (0.91–1.75) | |
| duration < 1 month | 0.96 (0.77–1.19) | 1.65 (0.92–2.98) | 1.20 (0.75–1.90) | |
| duration 1–6 month | 1.09 (0.90–1.31) | 1.07 (0.62–1.86) | 1.56 (1.07–2.28) | |
| duration ≥ 7 months | 0.91 (0.74–1.13) | 1.11 (0.59–2.10) | 0.94 (0.60–1.47) | |
| c) Test for trend with duration of breastfeeding⁺ | $\chi_1^2 = 0.52$ *P* = 0.47 | $\chi_1^2 = 0.82$ *P* = 0.36 | $\chi_1^2 = 2.63$ *P* = 0.11 | |

*Adjusted by age at diagnosis, sex, region, birth order and deprivation index, using as the control population children with other cancers. ⁺Excludes never breast fed and unknown duration categories, based on median duration within each duration category (11 days, 106 days and 330 days) for the control population (children with other cancers).

 *© 2001 Cancer Research Campaign*

**Figure 1** Odds ratio for childhood leukaemia by history of having been breastfed

| Study | Cases/Controls Never | Cases/Controls Ever | Odds ratio (95% CI) for ever versus never |
|---|---|---|---|
| van Steensel-Moll et al (1986) | 195/198 | 321/302 | 1.10 (0.80–1.40) |
| McKinney et al (1987)[a] | 85/264 | 78/235 | 1.02 (0.71–1.47) |
| Davis et al (1988)[b] | 16/74 | 47/107 | 2.03 (1.08–3.83) |
| Magnani et al (1988)[c,d] | 75/148 | 88/159 | 1.09 (0.75–1.60) |
| Shu et al (1995)[c,d] | 38/43 | 121/116 | 1.17 (0.70–1.90) |
| Petridou et al (1997) | 52/106 | 101/194 | 0.85 (0.52–1.41) |
| Dockerty et al (1999)[c,e] | 8/34 | 89/269 | 0.98 (0.39–2.47) |
| Schüz et al (1999)[c,d] | 439/409 | 562/592 | 0.88 (0.74–1.06) |
| Shu et al (1999)[c,d] | 1126/1096 | 1074/1322 | 0.79 (0.70–0.91) |
| Smulevich et al (1999)[c] | 40/55 | 159/343 | 0.64 (0.41–1.00) |
| Infante-Rivard et al (2000)[e] | 282/239 | 209/252 | 0.68 (0.49–0.95) |
| Rosenbaum et al (2000)[e,f] | 136/372 | 119/388 | 0.84 (0.63–1.11) |
| Bener et al (2001)[c,e] | 37/22 | 32/47 | 0.35 (0.21–0.62) |
| Hardell et al (2001)[c] | 30/31 | 205/206 | 0.90 (0.50–1.60) |
| UKCCS | 629/2500 | 1008/4464 | 0.89 (0.80–1.00) |
| **All studies** | **3188/5591** | **4213/8996** | **0.86 (0.81-0.92)** |

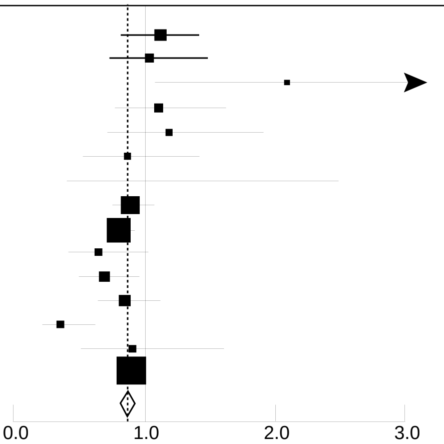

Test for heterogeneity: $\chi^2_{14}$=30.21; *P* < 0.01

Odds ratio for childhood leukaemia by duration of breastfeeding compared to never breastfed

| Study | Cases/Controls | Odds ratio (95% CI) versus never breastfed |
|---|---|---|
| **Duration ≤ 6 months** | | |
| McKinney et al (1987)[a] | 68/178 | 1.17 (0.80–1.71) |
| Davis et al (1988)[b] | 20/43 | 2.15 (1.02–4.55) |
| Magnani et al (1988)[c,d] | 73/131 | 1.10 (0.74–1.64) |
| Shu et al (1995)[c,d] | 37/33 | 1.26 (0.70–2.40) |
| Dockerty et al (1999)[c,e] | 47/123 | 1.24 (0.47–3.23) |
| Schüz et al (1999)[c,d] | 416/417 | 0.93 (0.77–1.13) |
| Shu et al (1999)[c,d] | 623/704 | 0.87 (0.75–1.01) |
| Smulevich et al (1999)[c] | 92/167 | 0.76 (0.47–1.22) |
| Infante-Rivard et al (2000)[e,g] | 104/125 | 0.68 (0.49–0.95) |
| Hardell et al (2001)[c] | 128/124 | 0.90 (0.50–1.70) |
| UKCCS | 705/3049 | 0.91 (0.80–1.03) |
| **All studies** | **2313/5094** | **0.91 (0.85–0.99)** |
| **Duration > 6 months** | | |
| McKinney et al (1987)[a] | 9/57 | 0.48 (0.23–1.03) |
| Davis et al (1988)[b] | 27/64 | 1.95 (0.97–3.91) |
| Magnani et al (1988)[c,d] | 15/28 | 1.06 (0.54–2.08) |
| Shu et al (1995)[c,d] | 84/83 | 1.14 (0.70–1.90) |
| Dockerty et al (1999)[c,e] | 40/146 | 0.62 (0.28–1.38) |
| Schüz et al (1999)[c,d] | 146/175 | 0.78 (0.60–1.01) |
| Shu et al (1999)[c,d] | 450/617 | 0.70 (0.59–0.82) |
| Smulevich et al (1999)[c] | 67/176 | 0.52 (0.32–0.86) |
| Infante-Rivard et al (2000)[e,g] | 105/127 | 0.67 (0.47–0.94) |
| Hardell et al (2001)[c] | 77/82 | 0.90 (0.50–1.70) |
| UKCCS | 302/1410 | 0.85 (0.73–1.00) |
| **All studies** | **1322/2965** | **0.78 (0.71-0.85)** |

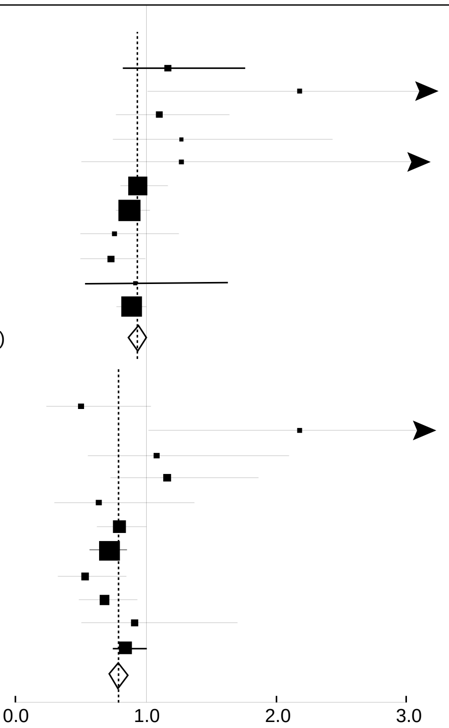

Test for heterogeneity ≤ 6 months: $\chi^2_{10}$ = 13.01; *P* > 0.1; NS

Test for heterogeneity > 6 months: $\chi^2_{10}$ = 17.92; *P* < 0.1

Test for heterogeneity between short-term and long-term breastfeeding: $\chi^2_{1}$ = 6.90; *P* < 0.01

[a]Data provided by McKinney (personal communication)
[b]Numbers of cases and controls derived from data presented by the authors
[c]"Never" includes breastfed for < 2 days (Dockerty et al 1999); < 1 month (Magnani et al 1988, Shu et al 1995, Schüz et al 1999, Shu et al 1999, Hardell et al 2001); < 2 months (Smulevich et al 1999); ≤ 6 months (Bener et al 2001)
[d]Acute leukaemia only
[e]Acute lymphatic leukaemia only
[f]Data from meeting abstract
[g]Duration ≤ 3 months and > 3 months

Hodgkin's lymphoma; 1.03 (0.68–1.58) for other childhood cancers; and 0.99 (0.72–1.36) for all cancers combined.

When children with other cancers are taken as the comparison group, the odds ratios associated with ever having been breastfed

© 2001 Cancer Research Campaign are 1.00, 1.24 and 1.26, respectively for leukaemia, Hodgkin's disease and non-Hodgkin's lymphoma (Table 5).

The results from the UKCCS study with respect to breast-feeding were compared with relevant results from other epidemiological studies of childhood cancer (van Steensel-Moll et al, 1986; McKinney et al, 1987; Davis et al, 1988; Hartley et al, 1988; Magnani et al, 1988; Golding et al, 1990; Shu et al, 1995; Petridou et al, 1997; Grufferman et al, 1998; Dockerty et al, 1999; Schüz et al, 1999; Shu et al, 1999; Smulevich et al, 1999; Infante-Rivard et al, 2000; Rosenbaum et al, 2000; Bener et al, 2001; Hardell and Dreifaldt, 2001). The UKCCS results did not differ significantly from those of previous studies, they have been combined with these and the overall results are shown for leukaemia (Figure 1), Hodgkin's disease (Figure 2), non-Hodgkin's lymphoma (Figure 3), other childhood cancers (Figure 4) and all childhood cancers combined (Figure 5). Not all studies published separate results for each cancer type and so the totals for each type do not add up to the 'all cancer' values. For childhood leukaemia, there is evidence of a statistically significant reduction in the odds ratio associated with ever having been breastfed (OR = 0.86, 95% CI 0.81–0.92) and having been breastfed for more than 6 months (OR = 0.78, 95% CI 0.71–0.85). However, there is also evidence of a reduction in the odds ratio of similar magnitude associated with ever having been breastfed for Hodgkin's disease (OR = 0.74, 95% CI 0.58–0.93), all non-haematological cancers (OR = 0.85, 95% CI 0.76–0.95) and for all childhood cancers combined (OR = 0.90, 95% CI 0.84–0.97).

### DISCUSSION

Breastfeeding provides a range of immunological benefits to the infant (Jelliffe and Jelliffe, 1978; Goldman, 1993; Cuthbertson, 1999; WHO Collaborative Team on the Role of Breastfeeding on the Prevention of Infant Mortality, 2000). In the first hours after birth, breast milk contains anti-microbial immunoglobulins; subsequently it also contains lymphocytes, macrophages and soluble factors, such as cytokines, lactoferrin, lysozyme, complement components, that may alter response to infection (Goldman, 1993; Hanson, 2000). However, breastfeeding also predisposes to the transmission of various infectious agents from mother to child (Ruff, 1994; Nduati et al, 2000).

Greaves' delayed exposure hypothesis predicts a protective effect for protracted breastfeeding specifically for childhood acute lymphoblastic leukaemia as a consequence of priming of the developing immune system by both vertical transmission of infective agents and immunological properties of breast milk (Greaves, 1997). The results from the UKCCS provide, however, equivocal

**Figure 2** Odds ratio for childhood Hodgkin's disease by history of having been breastfed

| Study | Cases/Controls Never | Cases/Controls Ever | Odds ratio (95% CI) for ever versus never |
|---|---|---|---|
| Davis et al (1988)[a] | 8/74 | 5/107 | 0.43 (0.14–1.31) |
| Shu et al (1995)[b] | 3/2 | 11/12 | 0.50 (0.10–5.50) |
| Grufferman et al (1998)[c] | 221/245 | 223/411 | 0.68 (0.50–0.94) |
| Smulevich et al (1999)[b] | 4/8 | 43/86 | 1.00 (0.30–3.30) |
| Bener et al (2001)[b] | 10/4 | 12/18 | 0.27 (0.07–1.01) |
| Hardell et al (2001)[b] | 7/6 | 21/24 | 0.50 (0.10–2.70) |
| UKCCS | 43/2500 | 71/4464 | 1.01 (0.67–1.53) |
| **All studies** | **296/2839** | **386/5122** | **0.74 (0.58–0.93)** |

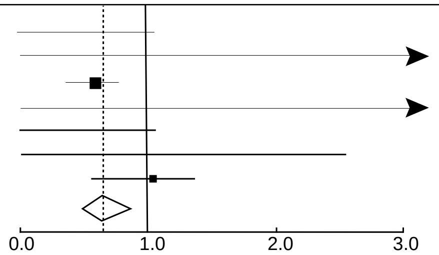

Test for heterogeneity: $\chi^2_6 = 6.16$; $P > 0.1$; NS

Odds ratio for childhood Hodgkin's disease by duration of breastfeeding compared to never breastfed

| Study | Cases/Controls | Odds ratio (95% CI) versus never breastfed |
|---|---|---|
| **Duration ≤ 6 months** | | |
| Davis et al (1988)[a] | 5/43 | 1.08 (0.35–3.35) |
| Shu et al (1995)[b] | 3/4 | 0.47 (0.00–5.80) |
| Smulevich et al (1999)[b] | 27/37 | 1.46 (0.42–5.03) |
| Hardell et al (2001)[b] | 13/14 | 0.60 (0.10–4.50) |
| UKCCS | 52/3049 | 1.06 (0.69–1.63) |
| **All studies** | **100/3147** | **1.06 (0.73–1.54)** |
| **Duration > 6 months** | | |
| Davis et al (1988)[a] | 0/64 | 0.00 (0.00–0.57) |
| Shu et al (1995)[b] | 8/8 | 0.57 (0.00–8.90) |
| Smulevich et al (1999)[b] | 16/49 | 0.65 (0.18–2.31) |
| Hardell et al (2001)[b] | 8/10 | 0.50 (0.10–2.80) |
| UKCCS | 19/1410 | 0.90 (0.51–1.62) |
| **All studies** | **51/1541** | **0.65 (0.40–1.06)** |

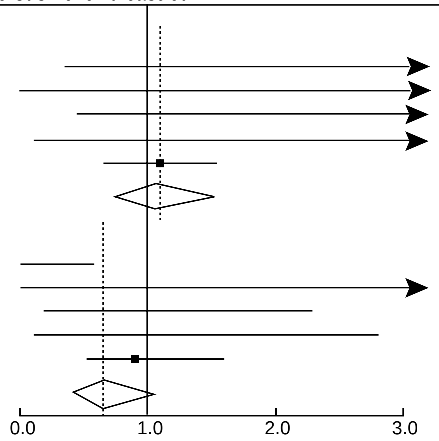

Test for heterogeneity ≤ 6 months: $\chi^2_4 = 0.80$; $P > 0.1$; NS
Test for heterogeneity > 6 months: $\chi^2_4 = 17.55$; $P < 0.01$
Test for heterogeneity between short-term and long-term breastfeeding: $\chi^2_1 = 2.44$; $P > 0.1$; NS

[a]Numbers of cases and controls derived from data presented by the authors
[b]"Never" includes breastfed for < 1 month (Shu et al 1995, Hardell et al 2001); < 2 months (Smulevich et al 1999); ≤6 months (Bener et al 2001)
[c]Data from meeting abstract

 *© 2001 Cancer Research Campaign*

**Figure 3** Odds ratio for childhood non-Hodgkin's lymphoma by history of having been breastfed

| Study | Cases/Controls Never | Cases/Controls Ever | Odds ratio (95% CI) for ever versus never |
|---|---|---|---|
| McKinney et al (1987)[a] | 16/264 | 9/235 | 0.56 (0.23–1.35) |
| Davis et al (1988)[b] | 5/74 | 8/107 | 1.11 (0.36–3.34) |
| Magnani et al (1988)[c] | 9/148 | 10/159 | 1.03 (0.42–2.55) |
| Shu et al (1995)[c] | 17/12 | 51/56 | 0.62 (0.30–1.50) |
| Smulevich et al (1999)[c] | 12/14 | 58/126 | 0.54 (0.24–1.21) |
| Bener et al (2001)[c] | 9/3 | 17/23 | 0.25 (0.06–0.99) |
| Hardell et al (2001)[c] | 3/12 | 49/36 | 5.50 (1.20–25.0) |
| UKCCS | 79/2500 | 149/4464 | 1.03 (0.77–1.38) |
| **All studies** | **150/3027** | **351/5206** | **0.90 (0.72–1.14)** |

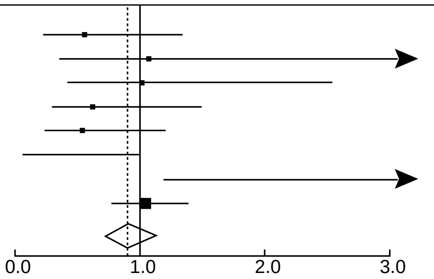

Test for heterogeneity: $\chi^2_7 = 13.17$; $P < 0.1$

Odds ratio for childhood non-Hodgkin's lymphoma by duration of breastfeeding compared to never breastfed

| Study | Cases/Controls | Odds ratio (95% CI) versus never breastfed |
|---|---|---|
| **Duration ≤ 6 months** | | |
| McKinney et al (1987)[a] | 6/178 | 0.49 (0.18–1.32) |
| Davis et al (1988)[b] | 6/43 | 2.07 (0.63–6.78) |
| Magnani et al (1988)[c] | 10/131 | 1.26 (0.51–3.10) |
| Shu et al (1995)[c] | 12/12 | 0.66 (0.20–2.40) |
| Smulevich et al (1999)[c] | 29/63 | 0.54 (0.22–1.29) |
| Hardell et al (2001)[c] | 29/21 | 5.10 (1.10–24.0) |
| UKCCS | 108/3049 | 1.09 (0.80–1.48) |
| **All studies** | **200/3497** | **1.04 (0.81–1.34)** |
| **Duration > 6 months** | | |
| McKinney et al (1987)[a] | 2/57 | 0.60 (0.13–2.78) |
| Davis et al (1988)[b] | 2/64 | 0.46 (0.00–2.15) |
| Magnani et al (1988)[c] | 0/28 | 0.00 (0.00–2.33) |
| Shu et al (1995)[c] | 39/44 | 0.61 (0.30–1.50) |
| Smulevich et al (1999)[c] | 29/63 | 0.54 (0.22–1.29) |
| Hardell et al (2001)[c] | 20/15 | 7.00 (1.30–37.0) |
| UKCCS | 41/1410 | 0.89 (0.60–1.34) |
| **All studies** | **133/1681** | **0.78 (0.57–1.07)** |

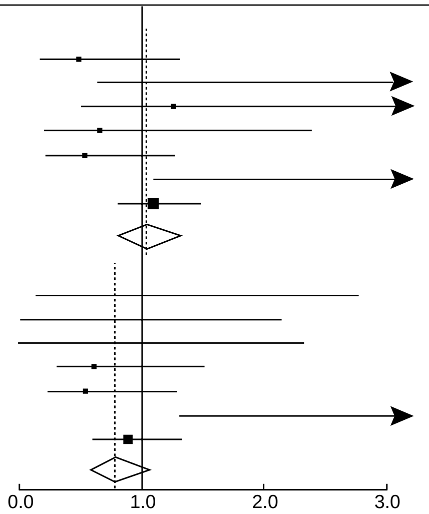

Test for heterogeneity ≤ 6 months: $\chi^2_6 = 10.46$; $P > 0.1$; NS
Test for heterogeneity > 6 months: $\chi^2_6 = 18.64$; $P < 0.01$
Test for heterogeneity between short-term and long-term breastfeeding: $\chi^2_1 = 1.89$; $P > 0.1$; NS

[a]Data provided by McKinney (personal communication)
[b]Numbers of cases and controls derived from data presented by the authors
[c]"Never" includes breastfed for < 1 months (Magnani et al 1988, Shu et al 1995, Hardell et al 2001); <2 months (Smulevich et al 1999); ≤ 6 months (Bener et al 2001)

support for the role of breastfeeding in the aetiology of childhood leukaemia. Although there is weak evidence, of borderline statistical significance, of a small reduction in the odds ratio for leukaemia in children who were breastfed, a reduction in the odds ratio of a similar magnitude was also found for all childhood cancers. Furthermore, the combined results from all relevant published studies suggest that, although having been breastfed is associated with an apparent small reduction in the odds ratio for childhood leukaemia (Figure 1), it is also associated with a small reduction in the odds ratio for Hodgkin's disease (Figure 2), non-haematological childhood cancers (Figure 4) and for all childhood cancers combined (Figure 5).

Information on breastfeeding practices were reported by mothers at the time of interview. Although there are known inaccuracies in such reporting (DHS Comparative Studies, 1999), it is unknown whether this is biased between the mothers of cases and controls. Studies comparing the duration of breastfeeding reported by mothers with the duration that was recorded at the time when their children were young indicate that women tend to round the reported durations to the nearest 6 months (DHS Comparative Studies, 1999).

Furthermore, 8 years after the birth of a child there is an overall tendency for women to report somewhat longer durations of breastfeeding than had been recorded at the time when the child was young (Vobecky et al, 1988). A review of the role of infant feeding in the aetiology of childhood diabetes mellitus showed that in studies where there was a substantially lower participation rate in controls than cases there was a tendency for the odds ratio associated with ever having been breastfed to be reduced (Norris and Scott, 1996). Participation in the UKCCS was lower in controls than cases (UK Childhood Cancer Study, 2000). Furthermore, participating controls were of higher socio-economic status than the first choice controls eligible for this study, but whether or not these factors produced bias is unknown.

In conclusion, it is unclear whether the apparent small reduction in the risk of each type of childhood cancer reported here is a non-specific effect of breastfeeding on childhood cancer or whether it reflects some systematic bias shared by the majority of the case-control studies that have investigated aetiological factors in

© 2001 Cancer Research Campaign

**Figure 4** Odds ratio for all childhood cancers except leukaemia and lymphoma by history of having been breastfed

| | Cases/Controls | | |
|---|---|---|---|
| **Study** | **Never** | **Ever** | **Odds ratio (95% CI) for ever versus never** |
| Davis et al (1988)[a] | 66/74 | 46/107 | 0.48 (0.30–0.78) |
| Smulevich et al (1999)[b] | 49/78 | 228/471 | 0.77 (0.52–1.14) |
| Hardell et al (2001)[a,b] | 78/67 | 423/459 | 0.79 (0.56–1.12) |
| UKCCS | 592/2500 | 929/4464 | 0.90 (0.80–1.02) |
| **All studies** | **785/2719** | **1626/5501** | **0.85 (0.76–0.95)** |

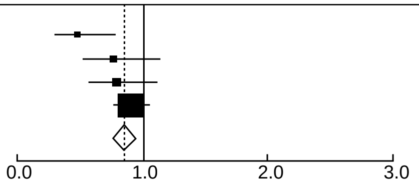

Test for heterogeneity: $\chi^2_3 = 6.76$; $P < 0.1$

Odds ratio for all childhood cancers except leukaemia and lymphoma by duration of breastfeeding compared to never breastfed

| **Study** | **Cases/Controls** | **Odds ratio (95% CI) versus never breastfed** |
|---|---|---|
| **Duration ≤ 6 months** | | |
| Davis et al (1988)[a] | 28/43 | 0.73 (0.41–1.30) |
| Smulevich et al (1999)[b] | 121/232 | 0.83 (0.55–1.26) |
| Hardell et al (2001)[a,b] | 239/273 | 0.75 (0.52–1.09) |
| UKCCS | 630/3049 | 0.89 (0.79–1.02) |
| **All studies** | **1018/3597** | **0.86 (0.77–0.97)** |
| **Duration > 6 months** | | |
| Davis et al (1988)[a] | 18/64 | 0.32 (0.17–0.58) |
| Smulevich et al (1999)[b] | 107/239 | 0.71 (0.47–1.09) |
| Hardell et al (2001)[a,b] | 184/186 | 0.85 (0.58–1.25) |
| UKCCS | 298/1410 | 0.93 (0.79–1.09) |
| **All studies** | **607/1899** | **0.85 (0.74–0.97)** |

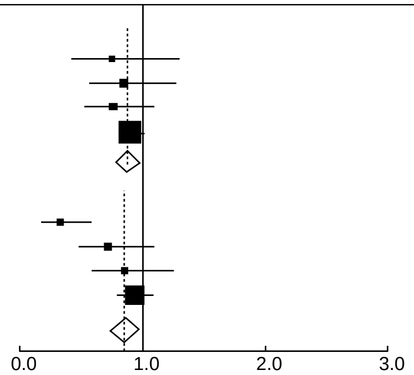

Test for heterogeneity ≤ 6 months: $\chi_3^2 = 1.13$; $P > 0.1$; NS
Test for heterogeneity > 6 months: $\chi_3^2 = 11.64$; $P < 0.01$
Test for heterogeneity between short-term and long-term breastfeeding: $\chi_1^2 = 0.05$; $P > 0.1$; NS
[a]Numbers of cases and controls derived from data presented by the authors
[b]"Never" includes breastfed for < 1 month (Hardell et al 2001); < 2 months (Smulevich et al 1999)

childhood cancer. We are unable to decide which of these possibilities is the more likely.

### ACKNOWLEDGEMENTS

The United Kingdom Childhood Cancer Study is sponsored and administered by the United Kingdom Co-ordinating Committee on Cancer Research and the Leukaemia Research Fund. The Study is conducted by 12 teams of investigators (10 clinical and epidemiological and 2 biological) based in university departments, research institutes and the National Health Service in Scotland. The work is coordinated by a Management Committee and in Scotland by a Steering Group. It is supported by the UK Children's Cancer Study Group of paediatric oncologists and by the National Radiological Protection Board. Financial support has been provided by the Cancer Research Campaign, the Imperial Cancer Research Fund, the Leukaemia Research Fund, and the Medical Research Council through grants to their units; by the Leukaemia Research Fund, the Department of Health, the Electricity Association, the Irish Electricity Supply Board (ESB), the National Grid Company plc, and Westlakes Research (Trading) Ltd through grants for the general expenses of the study; by the Kay Kendall Leukaemia Fund for the associated laboratories studies and by the Foundation for Children with Leukaemia for the study of electric fields. The investigation in Scotland is funded principally by The Scottish Office, and by ScottishPower plc, Scottish Hydro-Electric plc and Scottish Nuclear Ltd.

We should like to thank the members of the UK Childhood Cancer Study Group for their unstinting support and the staff of local hospitals, general practitioners and general practice staff, UKCCS interviewers and technicians, members of the electricity industry in England, Wales and Scotland and staff at schools for their invaluable help. We should especially like to thank the families of the children included in the study, without whom this investigation would not have been possible.

### APPENDIX

Management Committee: KK Cheng, Central region; NE Day, East Anglia region; R Cartwright and A Craft, North East region; JM Birch and OB Eden, North West region; PA McKinney, Scotland; J Peto, South East region; V Beral and E Roman, South Midlands region; P Elwood, South Wales region; FE Alexander, South West region; CED Chilvers, Trent region; R Doll, Epidemiological Studies Unit, University of Oxford, Oxford; CMTaylor, Immunogenetics Laboratory, University of Manchester, Manchester; M Greaves, Leukaemia Research Fund Centre, Institute of Cancer Research; D Goodhead, Radiation and Genome Stability Unit, Medical Research Council, Harwell; FA Fry, National Radiological Protection Board; G Adams, UK Coordinating Committee for Cancer Research.

Regional Investigators: KK Cheng and E Gilman, Central region; NE Day, J Skinner and D Williams, East Anglia region; R Cartwright and A Craft, North East region; JM Birth and OB Eden, North West region; PA McKinney, Scotland; J Deacon and J Peto, South East region; V Beral and E Roman, South Midlands region; P Elwood, South Wales region; FE Alexander and M Mott,

 *© 2001 Cancer Research Campaign*

**Figure 5** Odds ratio for all childhood cancers by history of having been breastfed

| Study | Cases/Controls Never | Cases/Controls Ever | Odds ratio (95% CI) for ever versus never |
|---|---|---|---|
| Davis et al (1988)[a] | 95/74 | 106/107 | 0.77 (0.51–1.16) |
| Hartley et al (1988) | 230/464 | 290/586 | 1.00 (0.80–1.24) |
| Golding et al (1990) | 24/53 | 9/46 | 0.43 (0.19–1.01) |
| Smulevich et al (1999)[b] | 105/155 | 488/1026 | 0.70 (0.54–0.92) |
| Hardell et al (2001)[b] | 119/116 | 716/744 | 1.00 (0.70–1.30) |
| UKCCS | 1343/2500 | 2157/4464 | 0.92 (0.84–1.00) |
| **All studies** | **1916/3362** | **3766/6973** | **0.90 (0.84–0.97)** |

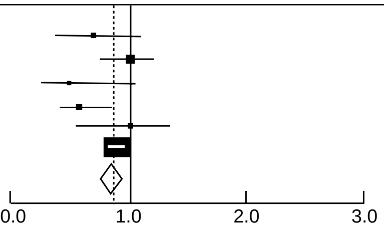

Test for heterogeneity: $\chi_5^2 = 8.54$; $P > 0.1$; NS

Odds ratio for all childhood cancers by duration of breastfeeding compared to never breastfed

| Study | Cases/Controls | Odds ratio (95% CI) versus never breastfed |
|---|---|---|
| **Duration ≤ 6 months** | | |
| Davis et al (1988)[a] | 59/43 | 1.07 (0.65–1.75) |
| Smulevich et al (1999)[b] | 269/499 | 0.80 (0.60–1.06) |
| Hardell et al (2001)[b] | 421/444 | 0.90 (0.70–1.30) |
| UKCCS | 1495/3049 | 0.92 (0.84–1.02) |
| **All studies** | **2244/4035** | **0.91 (0.84–0.99)** |
| **Duration > 6 months** | | |
| Davis et al (1988)[a] | 47/64 | 0.57 (0.35–0.93) |
| Smulevich et al (1999)[b] | 219/527 | 0.61 (0.46–0.82) |
| Hardell et al (2001)[b] | 295/300 | 1.00 (0.70–1.40) |
| UKCCS | 660/1410 | 0.90 (0.79–1.01) |
| **All studies** | **1221/2301** | **0.85 (0.76–0.94)** |

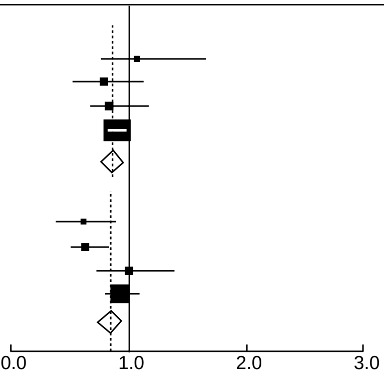

Test for heterogeneity ≤ 6 months: $\chi_3^2 = 1.25$; $P > 0.1$; NS
Test for heterogeneity > 6 months: $\chi_3^2 = 9.30$; $P < 0.05$
Test for heterogeneity between short-term and long-term breastfeeding: $\chi_1^2 = 1.15$; $P > 0.1$; NS
[a]Numbers of cases and controls derived from data presented by the authors
[b]"Never" includes breastfed for < 1 month (Hardell et al 2001); < 2 months (Smulevich et al 1999)

South West region; CED Chilvers and K Muir, Trent region; NT Fear, G Law, J Simpson and E Roman, Leukaemia Research Fund Data Management Processing Group.

### REFERENCES

Bener A, Denic S and Galadari S (2001) Longer breastfeeding and protection against childhood leukaemia and lymphomas. *Eur J Cancer* **37**: 234–238

Cuthbertson WFJ (1999) Evolution of infant nutrition. *Br J Nutr* **81**: 359–371

Davis MK, Savitz DA and Graubard BI (1988) Infant feeding and childhood cancer. *Lancet* **8607**: 365–368

DHS Comparative Studies (1999) Breastfeeding and complementary infant feeding, and the postpartum effects of breastfeeding. *Demographic and Health Surveys Comprehensive Studies No 30*, Macro International Inc, Maryland, USA

Dockerty JD, Skegg DCG, Elwood JM, Herbison GP, Becroft DMO and Lewis ME (1999) Infections, vaccinations, and the risk of childhood leukaemia. *Br J Cancer* **80**: 1483–1489

Epstein MA, Achong BG and Barr YM (1964) Virus particles in cultured lymphoblasts from Burkitt's lymphoma. *Lancet* **i**: 702–703

Golding J, Paterson M and Kinlen LJ (1990) Factors associated with childhood cancer in a national cohort study. *Br J Cancer* **62**: 304–308

Goldman AS (1993) The immune system of human milk: antimicrobial, antiinflammatory and immunomodulating properties. *Pediatr Infect Dis J* **12**: 664–672

Greaves MF (1997) Aetiology of acute leukaemia. *Lancet* **349**: 344–349

Greaves MF and Alexander FE (1993) An infectious etiology for common acute lymphoblastic leukemia in childhood? *Leukemia* **7**: 349–360

Gross L (1951) 'Spontaneous' leukemia developing in C3H mice following inoculation, in infancy, with AK-leukemic extracts, or AK-embryos. *Proc Soc Exp Biol Med* **76**: 27–32

Grufferman S, Davis MK, Ambinder RF, Shugart YY, Gilchrist GS and Brecher ML (1998) A protective effect of breastfeeding on risk of Hodgkin's disease in children. *Paediat Perinat Epidemiol* **12**: A13

Hanson LÅ (2000) The mother-offspring dyad and the immune system. *Acta Paediatr* **89**: 252–258

Hardell L and Dreifaldt AC (2001) Breastfeeding duration and the risk of malignant diseases in childhood in Sweden. *Eur J Clin Nutr* **55**: 179–185

Hartley AL, Birch JM, McKinney PA, Blair V, Teare MD, Carrette J, Mann JR, Stiller CA, Draper GJ, Johnston HE, Cartwright RA and Waterhouse JAH (1988) The Inter-Regional Epidemiological Study of Childhood Cancer (IRESCC): past medical history in children with cancer. *J Epidemiol Community Health* **42**: 235–242

Infante-Rivard C, Fortier I and Olson E (2000) Markers of infection, breast-feeding and childhood acute lymphoblastic leukaemia. *Br J Cancer* **83**: 1559–1564

Jelliffe DB and Jelliffe EFP (1978) *Human milk in the modern world*. Oxford: OUP

Kinlen LJ (1995) Epidemiological evidence for an infective basis in childhood leukaemia. *Br J Cancer* **71**: 1–5

Magnani C, Pastore G and Terracini B (1988) Infant feeding and childhood cancer. *Lancet* **2**: 1136

McKinney PA, Cartwright RA, Saiu JMT, Mann JR, Stiller CA, Draper GJ, Hartley AL, Hopton PA, Birch JM, Waterhouse JAH and Johnston HE (1987) The inter-regional epidemiological study of childhood cancer (IRESCC): a case-control study of aetiological factors in leukaemia and lymphoma. *Arch Dis Child* **62**: 279–287

Nduati R, John G, Mbori-Ngacha D, Richardson B, Overbaugh J, Mwatha A, Ndinya-Achola J, Bwayo J, Onyango FE, Hughes J and Kreiss J (2000) Effect of breastfeeding and formula feeding on transmission of HIV-1. A randomized clinical trial. *JAMA* **283**: 1167–1174

Norris JM and Scott FW (1996) A meta-analysis of infant diet and insulin-dependent diabetes mellitus: Do biases play a role? *Epidemiology* **7**: 87–92

*© 2001 Cancer Research Campaign*

Petridou E, Trichopoulos D, Kalapothaki V, Pourtsidis A, Kogevinas M, Kalmanti M, Koliouskas D, Kosmidis H, Panagiotou JP, Piperopoulou F and Tzortzatou F (1997) The risk profile of childhood leukaemia in Greece: a nationwide case-control study. *Br J Cancer* **76**: 1241–1247

Poiesz BJ, Ruscetti FW, Gazdar AF, Bunn PA, Minna JD and Gallo RC (1980) Detection and isolation of type C retrovirus particles from fresh and cultured lymphocytes of a patient with cutaneous T-cell lymphoma. *Proc Natl Acad Sci USA* **77**: 7415–7419

Rosenbaum PF, Buck GM and Brecher ML (2000) Breastfeeding and childhood acute lymphoblastic leukaemia. *Paediatr Perinat Epidemiol* **14**: A26

Ruff AJ (1994) Breast milk, breastfeeding, and transmission of viruses to the neonate. *Semin Perinatol* **18**: 510–516

Schüz J, Kaletsch U, Meinert R, Kaatsch P and Michaelis J (1999) Association of childhood leukaemia with factors related to the immune system. *Br J Cancer* **80**: 585–590

Shu X-O, Linet MS, Steinbuch M, Wen WQ, Buckley JD, Neglia JP, Potter JD, Reaman GH and Robison LL (1999) Breast-feeding and risk of childhood acute leukemia. *J Natl Cancer Inst* **91**: 1765–1772

Shu X-O, Clemens J, Zheng W, Ying DM, Ji BT and Jin F (1995) Infant breastfeeding and the risk of childhood lymphoma and leukaemia. *Int J Epidemiol* **24**: 27–32

Smulevich VB, Solionova LG and Belyakova SV (1999) Parental occupation and other factors and cancer risk in children: I. Study methodology and non-occupational factors. *Int J Cancer* **83**: 712–717

StataCorp (1999) Stata Statistical Software, release 6.0. College Station, TX, Stata Corporation

UK Childhood Cancer Study Investigators (2000) The United Kingdom Childhood Cancer Study: objectives, materials and methods. *Br J Cancer* **82**: 1073–1102

van Steensel-Moll HA, Valkenburg HA and van Zanen GE (1986) Childhood leukemia and infectious diseases in the first year of life: A register-based case-control study. *Am J Epidemiol* **124**: 590–594

Vobecky JS, Vobecky J and Froda S (1988) The reliability of the maternal memory in a retrospective assessment of nutritional status. *J Clin Epidemiol* **41**: 261–265

Vogt PK and Ishizaki R (1965) Reciprocal patterns of genetic resistance to Avian tumor viruses in two lines of chickens. *Virology* **26**: 664–672

WHO Collaborative Study Team on the Role of Breastfeeding on the Prevention of Infant Mortality (2000) Effect of breastfeeding on infant and child mortality due to infectious diseases in less developed countries: a pooled analysis. *Lancet* **355**: 451–455

*© 2001 Cancer Research Campaign*

## References

[PDF_00801] Bener A., Denic S., Galadari S. (2001). Longer breast-feeding and protection against childhood leukaemia and lymphomas.. Eur J Cancer.

[PDF_00803] Cuthbertson W. F. (1999). Evolution of infant nutrition.. Br J Nutr.

[PDF_00804] Davis M. K., Savitz D. A., Graubard B. I. (1988). Infant feeding and childhood cancer.. Lancet.

[PDF_00863] Davis N. A. (1988). Reproductive technologies and our attitudes towards children.. Logos (Santa Clara).

[PDF_00751] Davis R. B. (1988). Enhancing productivity in an integrated environment.. Comput Healthc.

[PDF_00765] Davis R. G. (1988). System design implications.. Comput Healthc.

[PDF_00809] Dockerty J. D., Skegg D. C., Elwood J. M., Herbison G. P., Becroft D. M., Lewis M. E. (1999). Infections, vaccinations, and the risk of childhood leukaemia.. Br J Cancer.

[PDF_00812] EPSTEIN M. A., ACHONG B. G., BARR Y. M. (1964). VIRUS PARTICLES IN CULTURED LYMPHOBLASTS FROM BURKITT'S LYMPHOMA.. Lancet.

[PDF_00822] GROSS L. (1951). "Spontaneous" leukemia developing in C3H mice following inoculation in infancy, with AK-leukemic extracts, or AK-embrvos.. Proc Soc Exp Biol Med.

[PDF_00814] Golding J., Paterson M., Kinlen L. J. (1990). Factors associated with childhood cancer in a national cohort study.. Br J Cancer.

[PDF_00816] Goldman A. S. (1993). The immune system of human milk: antimicrobial, antiinflammatory and immunomodulating properties.. Pediatr Infect Dis J.

[PDF_00819] Greaves M. F. (1997). Aetiology of acute leukaemia.. Lancet.

[PDF_00820] Greaves M. F., Alexander F. E. (1993). An infectious etiology for common acute lymphoblastic leukemia in childhood?. Leukemia.

[PDF_00828] Hanson L. A. (2000). The mother-offspring dyad and the immune system.. Acta Paediatr.

[PDF_00830] Hardell L., Dreifaldt A. C. (2001). Breast-feeding duration and the risk of malignant diseases in childhood in Sweden.. Eur J Clin Nutr.

[PDF_00833] Hartley A. L., Birch J. M., McKinney P. A., Blair V., Teare M. D., Carrette J., Mann J. R., Stiller C. A., Draper G. J., Johnston H. E. (1988). The Inter-Regional Epidemiological Study of Childhood Cancer (IRESCC): past medical history in children with cancer.. J Epidemiol Community Health.

[PDF_00838] Infante-Rivard C., Fortier I., Olson E. (2000). Markers of infection, breast-feeding and childhood acute lymphoblastic leukaemia.. Br J Cancer.

[PDF_00845] Magnani C., Pastore G., Terracini B. (1988). Infant feeding and childhood cancer.. Lancet.

[PDF_00847] McKinney P. A., Cartwright R. A., Saiu J. M., Mann J. R., Stiller C. A., Draper G. J., Hartley A. L., Hopton P. A., Birch J. M., Waterhouse J. A. (1987). The inter-regional epidemiological study of childhood cancer (IRESCC): a case control study of aetiological factors in leukaemia and lymphoma.. Arch Dis Child.

[PDF_00852] Nduati R., John G., Mbori-Ngacha D., Richardson B., Overbaugh J., Mwatha A., Ndinya-Achola J., Bwayo J., Onyango F. E., Hughes J. (2000). Effect of breastfeeding and formula feeding on transmission of HIV-1: a randomized clinical trial.. JAMA.

[PDF_00856] Norris J. M., Scott F. W. (1996). A meta-analysis of infant diet and insulin-dependent diabetes mellitus: do biases play a role?. Epidemiology.

[PDF_00913] Petridou E., Trichopoulos D., Kalapothaki V., Pourtsidis A., Kogevinas M., Kalmanti M., Koliouskas D., Kosmidis H., Panagiotou J. P., Piperopoulou F. (1997). The risk profile of childhood leukaemia in Greece: a nationwide case-control study.. Br J Cancer.

[PDF_00917] Poiesz B. J., Ruscetti F. W., Gazdar A. F., Bunn P. A., Minna J. D., Gallo R. C. (1980). Detection and isolation of type C retrovirus particles from fresh and cultured lymphocytes of a patient with cutaneous T-cell lymphoma.. Proc Natl Acad Sci U S A.

[PDF_00923] Ruff A. J. (1994). Breastmilk, breastfeeding, and transmission of viruses to the neonate.. Semin Perinatol.

[PDF_00925] Schüz J., Kaletsch U., Meinert R., Kaatsch P., Michaelis J. (1999). Association of childhood leukaemia with factors related to the immune system.. Br J Cancer.

[PDF_00931] Shu X. O., Clemens J., Zheng W., Ying D. M., Ji B. T., Jin F. (1995). Infant breastfeeding and the risk of childhood lymphoma and leukaemia.. Int J Epidemiol.

[PDF_00928] Shu X. O., Linet M. S., Steinbuch M., Wen W. Q., Buckley J. D., Neglia J. P., Potter J. D., Reaman G. H., Robison L. L. (1999). Breast-feeding and risk of childhood acute leukemia.. J Natl Cancer Inst.

[PDF_00934] Smulevich V. B., Solionova L. G., Belyakova S. V. (1999). Parental occupation and other factors and cancer risk in children: I. Study methodology and non-occupational factors.. Int J Cancer.

[PDF_00945] Vobecky J. S., Vobecky J., Froda S. (1988). The reliability of the maternal memory in a retrospective assessment of nutritional status.. J Clin Epidemiol.

[PDF_00947] Vogt P. K., Ishizaki R. (1965). Reciprocal patterns of genetic resistance to avian tumor viruses in two lines of chickens.. Virology.

[PDF_00942] van Steensel-Moll H. A., Valkenburg H. A., van Zanen G. E. (1986). Childhood leukemia and infectious diseases in the first year of life: a register-based case-control study.. Am J Epidemiol.

